# Bioconversion of biodiesel refinery waste in the bioemulsifier by *Trichosporon mycotoxinivorans* CLA2

**DOI:** 10.1186/1754-6834-5-29

**Published:** 2012-05-06

**Authors:** Andrea de Souza Monteiro, Vitor Souza Domingues, Marcus VD Souza, Ivana Lula, Daniel Bonoto Gonçalves, Ezequias Pessoa de Siqueira, Vera Lúcia dos Santos

**Affiliations:** 1 Departamento de Microbiologia, Instituto de Ciências Biológicas, Universidade Federal de Minas Gerais, C.P. 486, 31270-901, Belo Horizonte, MG, Brazil; 2 Laboratório de Pesquisa em Microbiologia – Faculdade de Ciências da Saúde, Universidade Vale do Rio Doce, Governador Valadares, MG, Brazil; 3 Laboratório de Encapsulamento Molecular e Biomateriais – LEMB, Dept. de Química, Instituto de Ciências Exatas e Tecnológicas, Universidade Federal de Minas Gerais, Belo Horizonte, MG, Brazil; 4 Laboratório de Ressonância Magnética Nuclear – Dept. de Química, Instituto de Ciências Exatas e Tecnológicas, Universidade Federal de Minas Gerais, Belo Horizonte, Brazil; 5 Laboratório de Processos Bioquímicos, Universidade Federal de São João Del-Rei, Campus Centro-Oeste Dona Lindu, Divinópolis, MG, Brazil; 6 Laboratório de Química de Produtos Naturais, Centro de Pesquisas René Rachou Fundação Oswaldo Cruz, 30190-002, Belo Horizonte, MG, Brazil

**Keywords:** Bioemulsifier, Yeasts, Response surface methodology, Biodiesel residue, Diatomaceous earth

## Abstract

**Background:**

The microbial bioemulsifiers was surface active compounds, are more effective in stabilizing oil-in-water emulsions. The yeasts have been isolated to produce bioemulsifiers from vegetable oils and industrial wastes.

**Results:**

*Trichosporon mycotoxinivorans* CLA2 is bioemulsifier-producing yeast strain isolated from effluents of the dairy industry, with ability to emulsify different hydrophobic substrates. Bioemulsifier production (mg/L) and the emulsifying activity (E_24_) of this strain were optimized by response surface methodology using mineral minimal medium containing refinery waste as the carbon source, which consisted of diatomaceous earth impregnated with esters from filters used in biodiesel purification. The highest bioemulsifier production occurred in mineral minimal medium containing 75 g/L biodiesel residue and 5 g/L ammonium sulfate. The highest emulsifying activity was obtained in medium containing 58 g/L biodiesel refinery residue and 4.6 g/L ammonium sulfate, and under these conditions, the model estimated an emulsifying activity of 85%. Gas chromatography and mass spectrometry analysis suggested a bioemulsifier molecule consisting of monosaccharides, predominantly xylose and mannose, and a long chain aliphatic groups composed of octadecanoic acid and hexadecanoic acid at concentrations of 48.01% and 43.16%, respectively. The carbohydrate composition as determined by GC-MS of their alditol acetate derivatives showed a larger ratio of xylose (49.27%), mannose (39.91%), and glucose (10.81%). ^1^ H NMR spectra confirmed by COSY suggested high molecular weight, polymeric pattern, presence of monosaccharide’s and long chain aliphatic groups in the bioemulsifier molecule.

**Conclusions:**

The biodiesel residue is an economical substrate, therefore seems to be very promising for the low-cost production of active emulsifiers in the emulsification of aromatics, aliphatic hydrocarbons, and kerosene.

## Background

Microbial bioemulsifiers are classified as high molecular weight surface-active compounds (SACs) that are effective in stabilizing oil-in-water emulsions [1]. Microorganisms synthesize a wide variety of high molecular weight SACs, which are composed of amphipathic polysaccharides, proteins, lipopolysaccharides, lipoproteins or complex mixtures of these biopolymers [2].

Microbial SACs have marked advantages over chemically synthesized surface-active compounds and can replace conventional surfactants in many areas, including agriculture and the textile, cosmetic and pharmaceutical industries [3]. In the environmental sector, microbial SACs have promising applications in bioremediation and waste treatment by participating in the removal of hazardous materials [2,4]. Their advantages over chemically synthesized compounds are numerous: they have many potential applications due to their novel structural characteristics and physical properties; they are produced from renewable substrates; they can be readily modified by genetic engineering, biologically or biochemically; and they are biodegradable [5].

Microbial SACs have recently been intensively studied because of their useful functional properties, such as emulsification, phase separation, wetting, foaming, solubilization, corrosion inhibition, and viscosity reduction [6,7]. High-molecular-weight SACs are produced by a wide variety of bacteria (Gram-positive and Gram-negative) and some strains of yeasts. Despite the production of bioemulsifiers by microorganisms, only a few strains of yeast have been described as bioemulsifier producers, including *Candida* and *Yarrowia*[8-11]. An example of a well-characterized high molecular weight SAC is Yansan, an effective emulsifier produced by *Yarrowia lipolytica*, IMUFRJ 50682, in a fermentation medium supplemented with glucose [11]. It was recently reported that yeast strains of the genus *Trichosporon* and *Geotrichum* have the ability to produce bioemulsifiers that are able to emulsify toluene. These SACs are produced in culture medium supplemented with sunflower oil, and the bioemulsifier compounds contain (9Z,12Z)-octadeca-9,12-dienoic acid and mannose, galactose, xylose and arabinose monosaccharides [12].

Despite such advantages, microbial SACs have not been commercialized due to their low yields and high cost of production. The cost of production is one of the principal factors to be considered in the development of any biotechnology process. While high production costs can be tolerated for microbial SACs used in low-volume applications, they are incompatible with applications that require high volumes of low-priced surfactants, such as enhanced oil recovery (EOR) [13,14]. During the last decade, many bioemulsifiers have been discovered from microorganisms using different substrates necessary for cell growth, such as carbohydrates, hydrocarbons, vegetable oils and glycerol [15].

Microbial SACs production largely depends, therefore, on the development of low cost processes, especially with regard to raw materials, because they represent 10-30% of the total production cost [6]. The goal of reducing the production cost of microbial SACs and environmental problems related to waste disposal has often been discussed in studies focusing on the production of these compounds from renewable sources. One of the main difficulties in the selection of alternative substrates includes finding the appropriate composition that allows cell growth and accumulation of the product of interest. In general, agroindustrial residues that contain high levels of carbohydrates or lipids serve as substrates for the production of microbial bioemulsifier SACs [16,17]. Waste generated from oil and biodiesel processing contains mainly organic compounds and may be an excellent candidate for use as a substrate for microbial growth [18,19].

During biodiesel oil production, the waste generated is primarily composed of methanol and crude glycerin, which are extracted during the transesterification process. After the subsequent deacidification and purification steps, filter materials, such as diatomaceous earth, are generated to adsorb residual oils and fatty acid methyl esters. The filter material has a high content of organic matter, and pre-treatment is necessary for subsequent disposal in the environment [20]. However, the compounds adsorbed on the filter can be used to support microbial growth and serve as a precursor for the production of microbial SACs, such as biosurfactants and bioemulsifiers. To our knowledge, there have been few attempts reported of using agroindustrial residues for bioemulsifier production, and only a few types of bioemulsifiers produced from agroindustrial residues have been described.

*Trichosporon mycotoxinivorans* CLA2 is basidiomycetic yeast and is characterized by the production of arthroconidia. *T. mycotoxinivorans* CLA2 is a bioemulsifier-producing yeast strain isolated from effluents of the dairy industry, with ability of emulsifying different hydrophobic substrates. The yeast CLA2 does not ferment glucose and has no growth at 40°C, but is able to grow in mineral medium containing sunflower oil in studies conducted by our group [12]. In this study, we applied response surface methodology (RSM) to optimize emulsifying activity and lipid-polysaccharide bioemulsifier production from this yeast. The experimental study was conducted by employing the statistical central composite rotatable design (CCRD) with three factors and five levels, including six replicates at the center point. The factors analyzed were concentrations of yeast extract, biodiesel residue (adsorbed on diatomaceous earth) and ammonium sulfate.

## Results and discussion

### Screening strains for emulsifying ability

A total of 30 strains of yeasts were tested for emulsification ability after inoculation in minimum medium (MM) supplemented with biodiesel refinery residue. Three of the evaluated strains exhibited emulsifying abilities, producing a cell-free supernatant that retained the capacity to emulsify kerosene after 4 weeks (data not shown). Using the screening process, we selected the strain CLA2, which was able to grow in MM medium containing 20 g/L biodiesel residue and exhibited an emulsifying activity (E_24_) for kerosene of around 60.4% after 144 hours of growth. The strain CLA2 was previously identified as *T. mycotoxinivorans* (the GenBank nucleotide sequence accession number is GU299455) [12].

### Optimization of emulsifying activity and bioemulsifier production

To evaluate the effect of three independent variables on the emulsifying activity and production of the bioemulsifier by *T. mycotoxinivorans* CLA2, a CCRD methodology was employed to evaluate the coefficients in a quadratic mathematical model. RSM was used to calculate the maximum production based on a few sets of experiments in which all the factors were varied within chosen ranges.

Under the conditions used in the CCRD, the bioemulsifier production ranged from 0 g/L to 7.077 mg/ml, and the emulsifying activity ranged from 0 to 82%. The central points for both responses revealed only small variations, indicating good reproducibility of the process. Table 1 presents the CCRD matrix with the levels of variables chosen for trials in the central composite design. Twenty experimental runs with different combinations of three factors were performed for each of the three repetitions. Table 1 also shows the observed and expected values of bioemulsifier production and emulsifying activity. The expected values were obtained using the polynomial equations model, based on regression coefficient findings.

**Table 1:** Experimental design and results of the CCRD matrix with observed and expected values of the bioemulsifier production and emulsifying activity

**Run**	**Variables/levels**			**Biemulsifier production (mg/l) (*Y***_**1**_**)**		**Emulsifying activity (E**_**24**_**) (*Y***_**2**_**)**	
	**Yeast extract (g/L) (*X***_**1**_**)**	**Residue biodiesel (g/L) (*X***_**2**_**)**	**Ammonium sulfate (g/L) (*X***_**3**_**)**	**Observerd**	**Expected**	**Observed**	**Expected**
1	−1.00	−1.00	−1.00	665.00	596.38	42.67	24.75
2	1.00	−1.00	−1.00	727.33	596.38	45.00	24.75
3	−1.00	1.00	−1.00	783.33	923.29	45.00	45.17
4	1.00	1.00	−1.00	841.33	923.29	45.00	45.17
5	−1.00	−1.00	1.00	3488.00	2908.37	78.33	63.07
6	1.00	−1.00	1.00	4458.67	2908.37	61.50	63.07
7	−1.00	1.00	1.00	6981.67	6174.95	61.94	83.49
8	1.00	1.00	1.00	7076.67	6174.95	80.67	83.49
9	−1.68	0.00	0.00	3517.33	2650.75	81.33	66.62
10	1.68	0.00	0.00	3859.00	2650.75	80.00	66.62
11	0.00	−1.68	0.00	0.00	1139,87	0.00	33.52
12	0.00	1.68	0.00	3523.00	4161.63	79.33	67.86
13	0.00	0.00	−1.68	0.00	−529.37	0,00	14.97
14	0.00	0.00	1.68	4064.67	5830.87	72.33	79.41
15	0.00	0.00	0.00	2194.67	2650.75	57.67	66.62
16	0.00	0.00	0.00	2205.67	2650.75	60.00	66.62
17	0.00	0.00	0.00	2189.33	2650.75	57.67	66.62
18	0.00	0.00	0.00	2145.67	2650.75	58.67	66.62
19	0.00	0.00	0.00	2159.00	2650.75	60.00	66.62
20	0.00	0.00	0.00	2134.67	2650.75	56.60	66.62

Values represent means of three experimental repetitions.

Through multiple regression analysis of the experimental data, a second-order polynomial equation was obtained for bioemulsifier production (Equation 1) and emulsifying activity (Equation 2).

(1) $$Y_{1}=53.6818-25.0418X_{2}+299.589X_{3}+26.2696X_{2}*X_{3}$$

(2) $$Y_{2}=-37.7272+1.63938X_{2}+32.3493X_{3}-0.0141048X_{2}^{2}-3.50229X_{3}^{2}$$

The statistical significance of Equations 1 and 2 was verified by the F test and by analysis of variance of the quadratic model of the RSM and is shown in Table 2. At a 95% confidence level, the models for bioemulsifier production and emulsifying activity present a calculated F-value higher than the table value [21], showing that the regression models were significant.

**Table 2:** **Analysis of variance (ANOVA) for the response of bioemulsifier production (*Y***_***1***_**)**^**a**^**and emulsifying activity ( *Y ***_***2***_**)**^**b**^**of *T. mycotoxinivorans *cultivated in medium containing biodiesel refinery residue**

**Response**	**Terms of the Model**	**Degrees of freedom**	**Estimated**	**Sum of squares**	**Mean squares**	***F-value***	***P-value***
*Y*_1_	b_0_	-	53.6818	-	-	-	0.000
	*X*_2_	1	−25.0418	33066316	33066316	47.56	0.000^c^
	*X*_3_	1	299.589	146492422	146492422	210.73	0.000^c^
	*X*_2_*X_3_	1	26.2696	12962460	12962460	18.65	0.000^c^
	Full model	3	-	192521198	64173733	92.31	0.000^c^
	Total Error	56	-	38930197	695182	-	-
	Total	59	-	-	-	-	-
*Y*_2_	b_0_	-	−37.7272	-	-	-	0.000
	*X*_2_	1	1.63938	4270.1	4270.1	22.21	0.000^c^
	*X*_3_	1	32.3493	15038.7	15038.7	78.22	0.000^c^
	*X*_2_^2^	1	- 0.0141048	1384	1384.0	7.20	0.010^c^
	*X*_3_^2^	1	- 3.50229	2059	2059.2	10.71	0.002^c^
	Full model	4	-	22473	5618.2	29.22	0.003^c^
	Total Error	55	-	10574	192.2	-	-
	Total	59	-	-	-	-	-

Coefficient of variation (CV): ^a^ 31.45, ^b^ 23.87. Coefficient of determination (*R*^*2*^): ^a^ 83.18_,_^b^ 68.00. ^c^ Significant at 5% level (*P* < 0.05).

The significance of the coefficients of the full second-order polynomial model for bioemulsifier production and emulsifying activity were evaluated by Student’s *t*-test and the *p*-values (Table 2). The *p*-values lower than 0.05 indicate that there is a significant correlation between the coefficients. The statistical significance of Eq. (1) and Eq. (2) was checked by the F-test, and the analysis of variance (ANOVA) for the response surface quadratic model is shown in Table 2.

For both analyses, the *X*_1_ factor (yeast extract) was not significant. On the other hand, the *X*_2_ (biodiesel residue) and *X*_3_ ((NH_4_)_2_SO_4_) factors were highly significant. For bioemulsifier production, the *X*_2_ * *X*_3_ interaction was significant, exhibiting a synergistic effect on bioemulsifier production. The coefficient of the linear term *X*_2_ was negative, which means that if there were no effects of the *X*_2_ * *X*_3_ interaction, increases in the levels of *X*_2_ would result in decreased bioemulsifier production.

For the emulsifying activity, it was found that the second order parameters of the model represented by *X*_1_, *X*_2_ and *X*_3_, the coefficients of the linear terms, were all positive, meaning that a higher emulsifying activity should be obtained by increasing the levels of each of the factors. However, because the coefficients of the quadratic terms are all negative, increases in the levels of the factors will also tend to decrease the response. This indicates that this model reached the optimum region of emulsifying activity.

The coefficient of variation (CV) was reasonable for both models (31.45 for bioemulsifier production; 23.87 for emulsifying activity), indicating good precision and reliability of the experiments. The precision of the models was determined by the determination coefficient (*R*^2^). The *R*^2^ value implies that the sample variation of 83.18% for bioemulsifier production and 68% for emulsifying activity were attributed to the independent variables, and only about 16.82% and 32%, respectively, of the total variation cannot be explained by the models.

**Figure 1:**
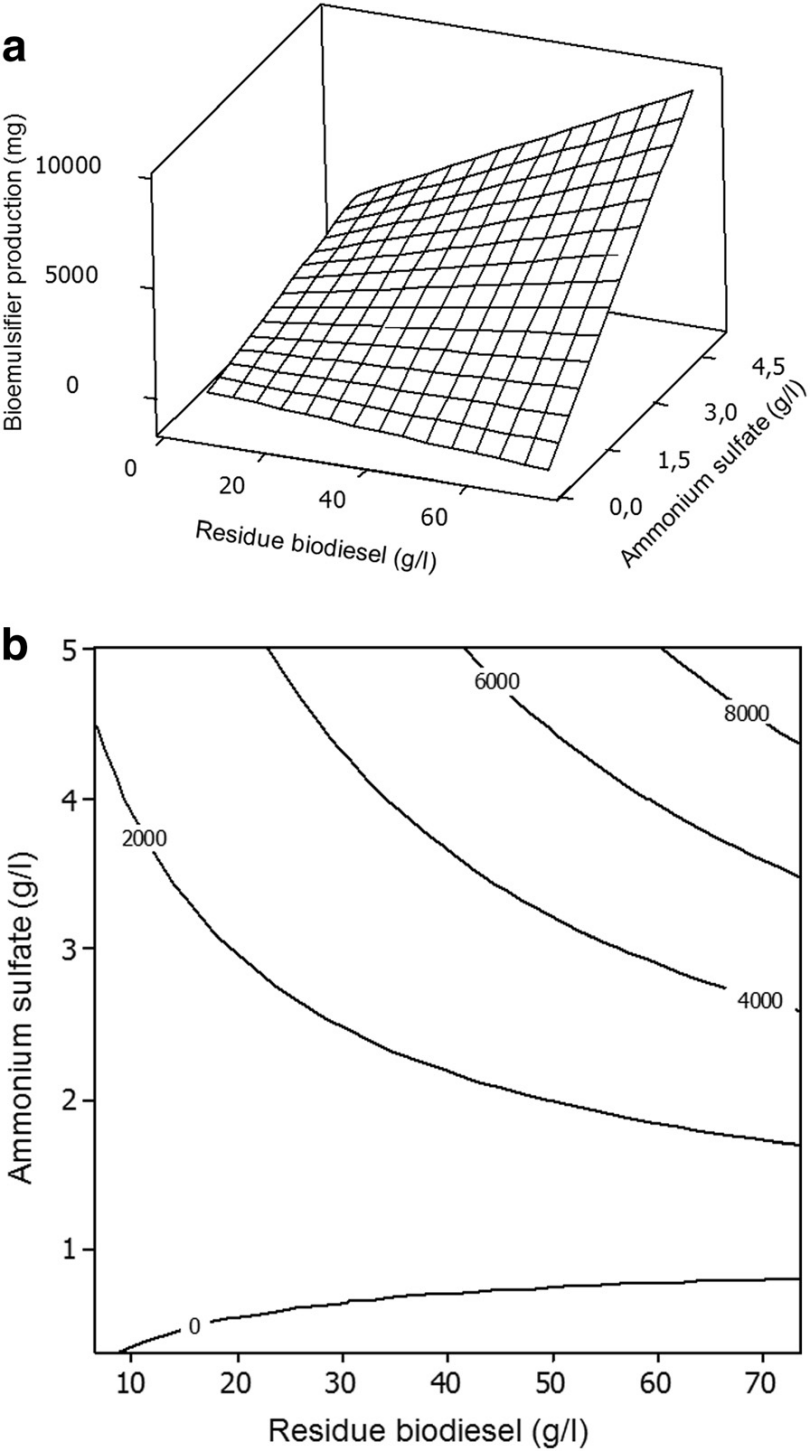
**a-b. Response surface plot and contour plot of the combined effects of residue biodiesel and ammonium sulfate on the bioemulsifier production by *T. mycotoxinivorans *CLA2 in a constant cultivation time (24 hours).**

**Figure 2:**
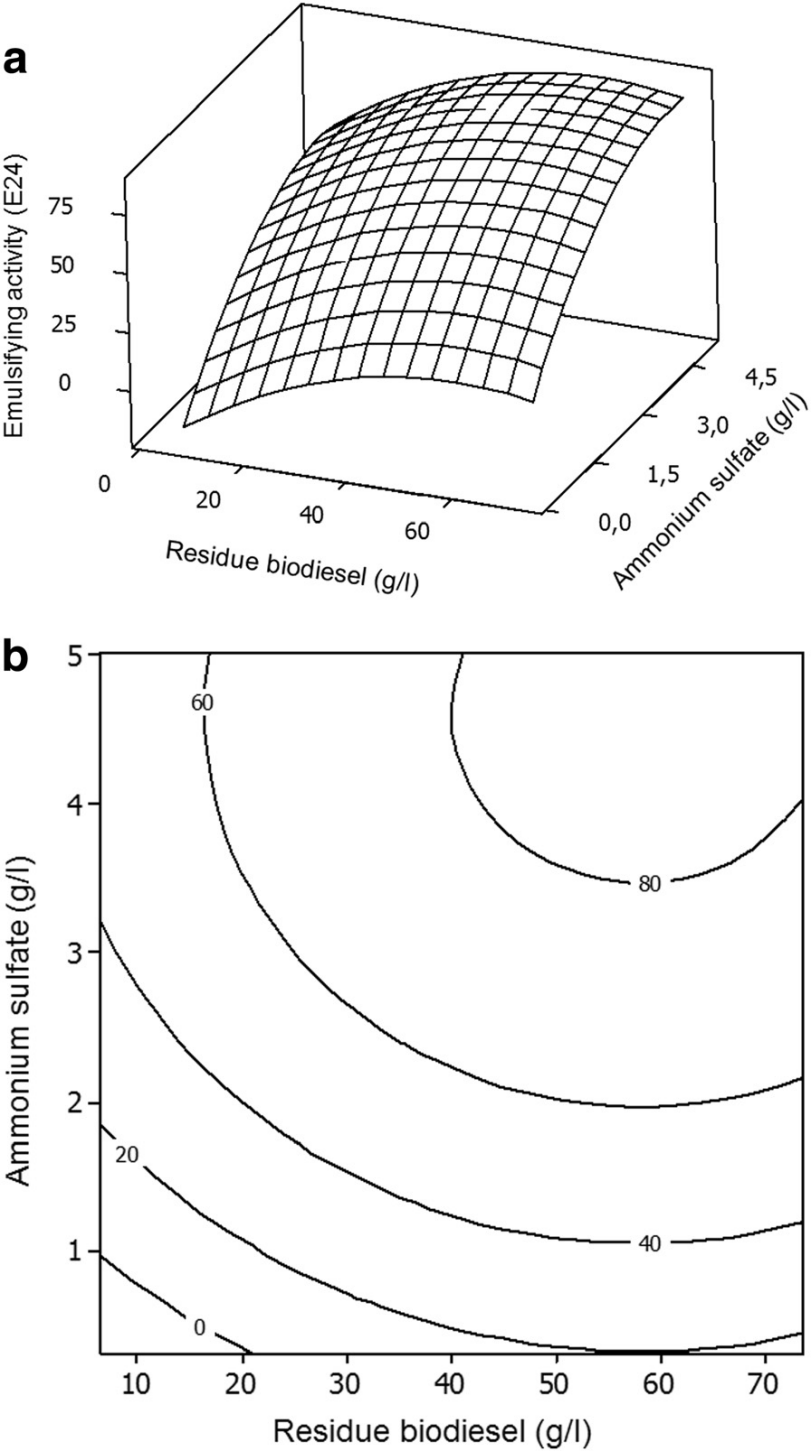
**a-b. Response surface plot and contour plot of the combined effects of residue biodiesel and ammonium sulfate on the emulsifying activity by *T. mycotoxinivorans *CLA2 in a constant cultivation time (24 hours).**

The response surfaces plots described by the regression models are presented in Figures 1a and 2a. The figures provide a surface three-dimensional visualization of the estimated trend for the variation of bioemulsifier production and emulsifying activity, respectively, by *T. mycotoxinivorans* CLA2, with different concentrations of biodiesel residue and ammonium sulfate. Although Figure 1a shows that the optimal region of the response has not yet been obtained, it indicates the parameters necessary for optimization. Figure 1b facilitates the identification of the maximum bioemulsifier production point within the range studied, located at a level delimited by 60.5 and 75 g/L biodiesel residue and 4.4 and 5 g/L ammonium sulfate. The estimated maximum bioemulsifier production occurred at 75 g/L biodiesel residue and 5 g/L ammonium sulfate. Under these conditions, the estimated bioemulsifier production was 9.525 mg.

Figure 2a provides evidence that the model encompassed the optimum region for emulsifying activity, located at the surface peak. Figure 2b shows that the range of the estimated highest emulsifying activity was located at a level delimited by 40 and 75 g/L biodiesel residue and 3.4 and 5 g/L ammonium sulfate. The estimated maximum emulsifying activity occurred at 58 g/L biodiesel residue and 4.6 g/L ammonium sulfate. Under these conditions, the estimated emulsifying activity was 85% (E_24_).

In the contour plots (Figures 1b and 2b), it is possible to visualize whether the mutual interactions between the independent variables are significant or not. Clearly, the fitted model for bioemulsifier production presents interactions between independent variables, due an elliptical nature of the contour plot.

RSM is the most accepted statistical analysis for bioprocess optimization, allowing the relationship between a set of experimental factors and observed results to be examined [22]. With regard to the production of microbial SACs as biosurfactants, the RSM has been successfully employed to optimize the production of these compounds from waste agricultural industries and to reduce the amount of organic material to be released in effluents [23,24]. Furthermore, other products, such as cheese, whey and molasses, are added to microbial growth media as an alternative to reduce the cost of producing microbial SACs using probiotic bacteria [25].

The residue derived from the production processes and purification of biodiesel consisting of organic compounds impregnated in diatomaceous earth was successfully employed to produce bioemulsifiers by *T. mycotoxinivorans* CLA2. The residue used has an organic matter concentration of 24.78%, composed mainly of fatty acids methyl esters and glycerol byproducts, which is capable of supporting the growth of microbial cells, without adding other carbon sources. In this case, the reduction of bioemulsifier production costs by employing biodiesel processing waste would be an advantage for the production of these compounds by yeasts. Due to concerns about the disposition of the diatomaceous earth adsorbents filters and because some landfills are refusing this waste due to the concern of spontaneous combustion [20], the microbial degradation of the compounds adsorbed in filters is advantageous and of great interest. It can eliminate the dangers of explosion and environmental contamination.

The described metabolic potential of the *Trichosporon* species has made the application of these yeasts for environmental purposes very promising [26]. Recently, a strain of *T. mycotoxinivorans* was described as promising for the detoxification of effluents containing mycotoxins, especially the cleavage of zearalenone [27]. Additionally, strains of the genus *Trichosporon* are capable of producing bioemulsifiers from the metabolism of sunflower oil, which is added as the sole carbon source for cellular growth [12]. To our knowledge, this is the first report on bioemulsifier production by *T. mycotoxinivorans* from growth medium supplemented with residue from the processing of biodiesel.

### Emulsifying activity of the bioemulsifier using hydrophobic substrates

**Figure 3:**
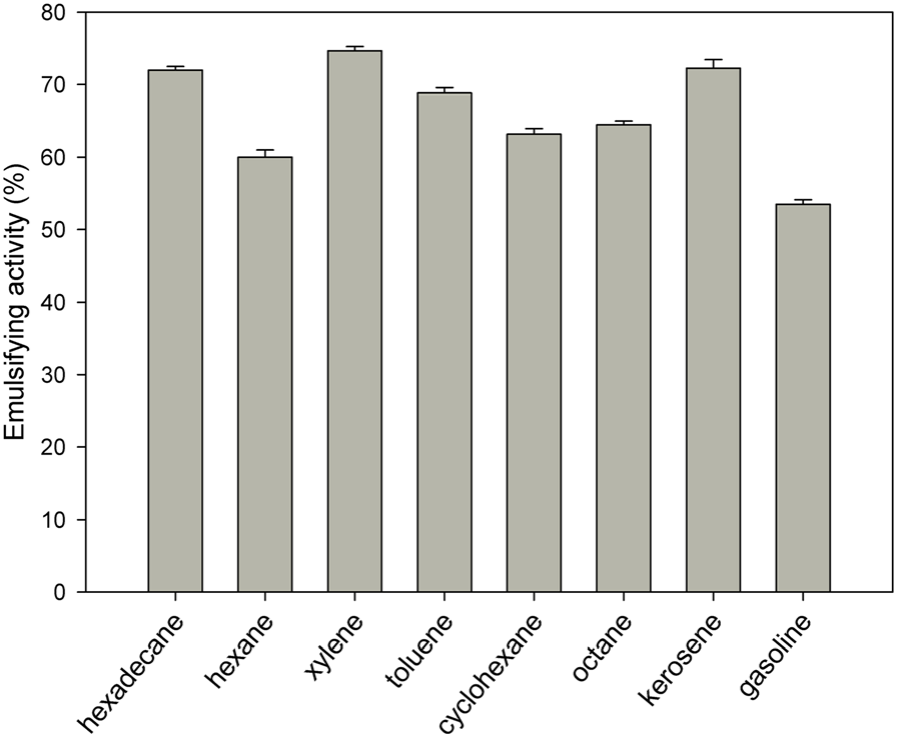
**Emulsifying activity (E**_**24**_**) of the crude bioemulsifier produced by *T. mycotoxinivorans *CLA2 using different hydrophobic substrates.**

In addition to the measure of surface tension, the stabilization of an oil/water emulsion is commonly used as surface activity indicator. The emulsifier specificity (5 mg/mL) produced by *T. mycotoxinivorans* CLA2 was assayed with various hydrophobic substrates (Figure 3). A high emulsifying activity value, approximately around 75%, was obtained using xylene. The emulsifying activities for toluene, hexadecane, and kerosene were approximately 71.7%. Statistical analysis of the test data by the Duncan test with 5% probability showed no significant differences between their values. Nevertheless, significant differences were observed between the values of emulsifying activity obtained for hexane, octane, cyclohexane, and gasoline.

Several studies have shown that biosurfactants and bioemulsifiers may vary in their ability to emulsify different hydrophobic compounds. It was suggested that the emulsifying activity depends on the bioemulsifier’s affinity for hydrocarbons substrates, which involves a direct interaction with the hydrocarbon itself, rather than an effect on the surface tension of the medium [28]. Poor emulsification of some hydrocarbons might be due to the inability of the bioemulsifier to stabilize the microscopic droplets. The broad-spectrum emulsifying activity is essential for the use of a bioemulsifier in industrial processes, such as the treatment of industrial effluents, washing of oily deposits and pumping of heavy oils, considering that the processes have different mixtures of hydrophobic compounds.

The bioemulsifier produced by *T. mycotoxinivorans* CLA2 from biodiesel residue appears to be a very effective and efficient emulsifier for aromatic and aliphatic hydrocarbons. Emulsan, one of the most effective emulsifiers, stabilizes hydrocarbon emulsions in water emulsions (the percentage of hydrocarbons vary from 0.01-0.10%) at low concentrations (0.02 to 0.2 mg/mL), and it exhibits considerable substrate specificity but does not emulsify pure aliphatic, aromatic or cyclic hydrocarbons. Nevertheless, all mixtures that contain an appropriate mixture of aliphatic and aromatic (or cyclic alkane) residues are efficiently emulsified [29].

### Purification and characterization of the bioemulsifier

Purification of the bioemulsifier produced by *T. mycotoxinivorans* CLA2 was performed by gel filtration chromatography. Fractionation of the bioemulsifier resulted in only one peak, with the resulting fractions being positive for total carbohydrate and emulsifying activity. The final purified yield of the bioemulsifier, based on the recovered mass fractionation, was 90% (data not shown).

The analysis of the fatty acid methyl esters of the lipid portion of the bioemulsifier indicated the presence of octadecanoic acid, hexadecanoic acid and 9-octadecenoic acid (Z) at concentrations of 48.01%, 43.16% and 8.83%, respectively. The content of fatty acids of this bioemulsifier resembles that of a high-molecular-weight bioemulsifier produced by bacteria and yeast, in which the highest percentage of fatty acids contain chains with 12, 16 and 18 carbons. [11] studying the bioemulsifier Yansan, observed that the fatty acid content corresponds mainly to fatty acids of 16 and 18 carbons, such as hexadecanoic acid (35. 8%) and octadecanoic acid (21.4%). However, the number of carbons in fatty acid chains of the bioemulsifier Emulsan produced by *Acinetobacter calcoaceticus* RAG-1 seems to be dependent on the type and combination of hydroxy and hydroxylated fatty acids used for microbial growth. Therefore, different structures of the bioemulsifier can be obtained by varying the type of the precursor [30].

Monosaccharides in the bioemulsifier were identified by GC–MS analysis of their alditol acetate derivatives. The analysis showed the presence of xylose (49.27%), mannose (39.91%) and glucose (10.81%). The composition of monosaccharides in the bioemulsifier from CLA2 is similar to that described for the bioemulsifier produced by *T. loubieri* CLV20, which contains mannose and glucose at concentrations of 41% and 8%, respectively [12]. Additionally, this composition is similar to that described for a biosurfactant produced by *Y. lipolytica* IMUFRJ 50682, which showed a predominance of mannose [11].

The high-molecular-weight SACs are not effective in reducing surface and interfacial tension of liquids but are very effective in stabilizing oil in water emulsions [31]. Generally, this hydrophobic portion is required for emulsification, such as in the emulsifiers produced by *Acinetobacter calcoaceticus* BD4 and *A. calcoaceticus* RAG-1 [29,32]. Similarly, the SACs produced by the yeast *T. mycotoxinivorans* CLA2 exhibited the ability to stabilize emulsions with kerosene as the organic phase. However, the bioemulsifier is not capable in significantly reducing the surface tension of water (data not shown).

### Characterization of the bioemulsifier by nuclear magnetic resonance (^1^ H NMR)

**Figure 4:**
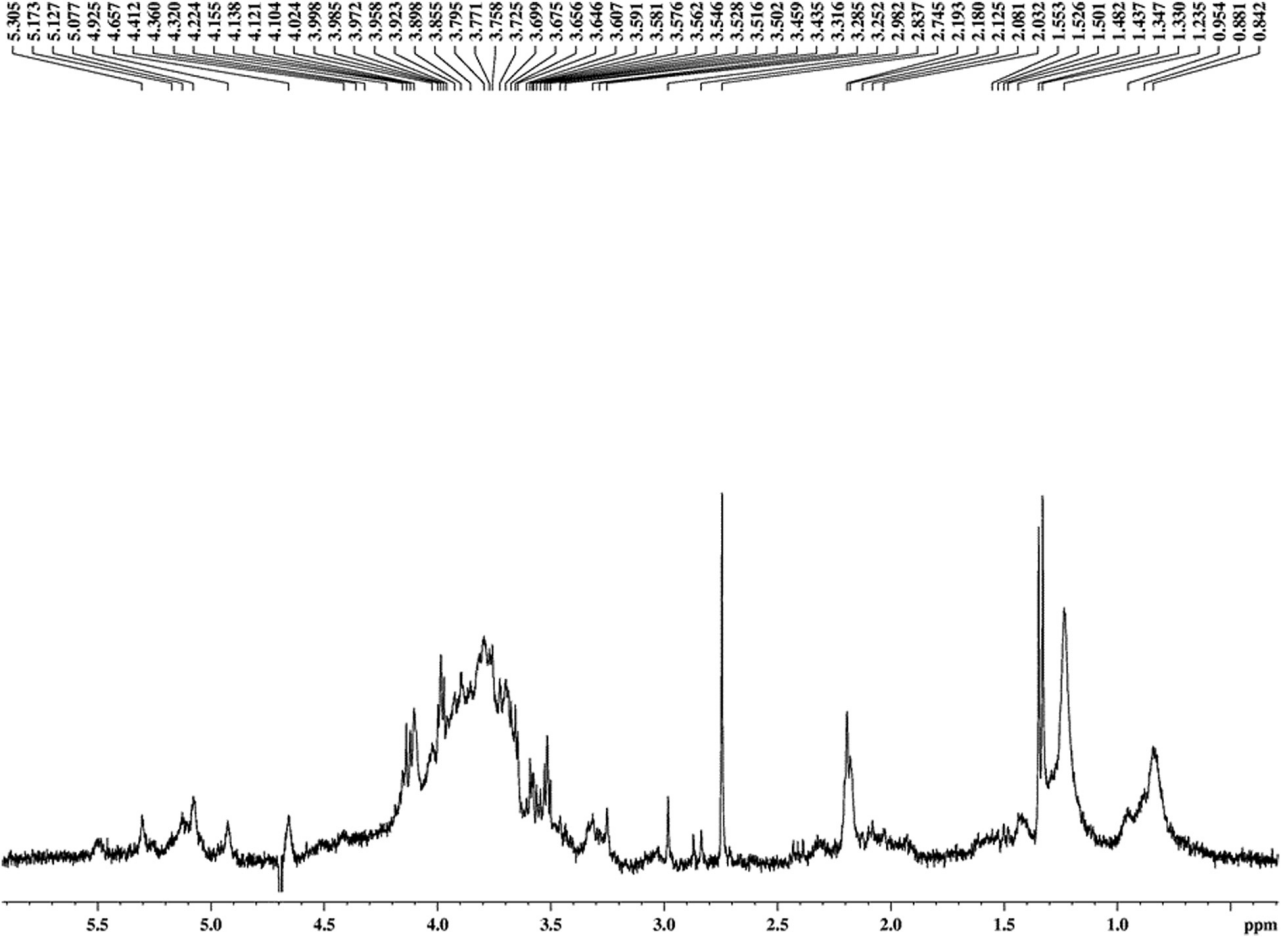
^**1**^**H nuclear magnetic resonance (NMR) spectrum of the bioemulsifier produced by *T. mycotoxinivorans *CLA2 at 27°C (presaturation, 400 MHz, D**_**2**_**O).**

**Figure 5:**
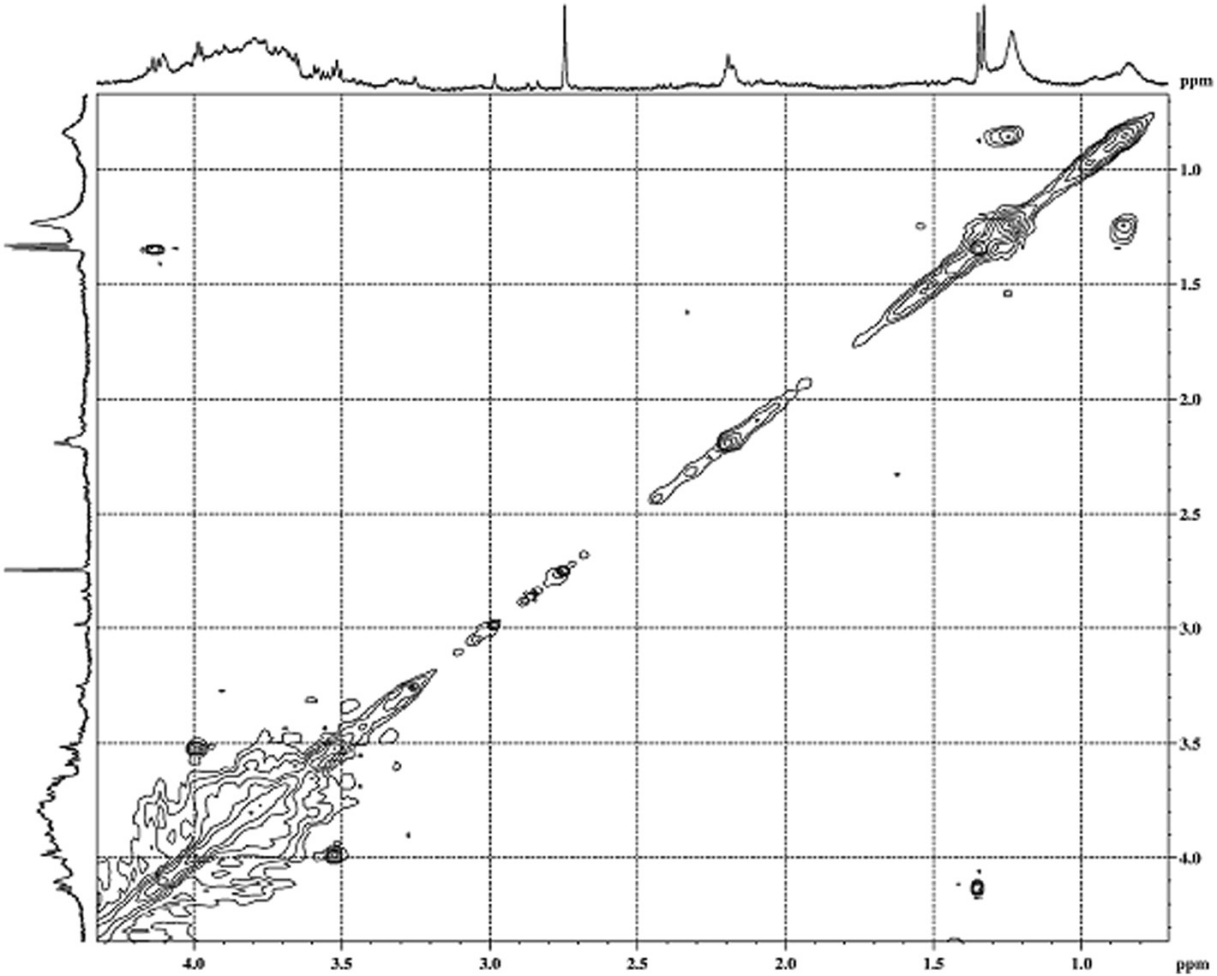
**COSY contour map of the bioemulsifier produced by *T. mycotoxinivorans *CLA2 at 27°C (400 MHz, D**_**2**_**O).** Scalar correlations between the polysaccharides group of the molecule and the hydrophobic side chains are indicated.

The results obtained with a 400 MHz ^1^ H NMR spectrum of the bioemulsifier molecule in D_2_O are shown in Figure 4. The high overlap of the signals only allows characteristic regions of chemical shifts of the hydrogen signals in saccharide groups to be identified. The ^1^ H NMR signals related to the anomeric hydrogens of the saccharide residues present in the polymer molecule appear at δ 4.6 and δ 5.5 ppm, with higher intensities at δ 5.05 ppm, indicating a higher proportion of this sugar in the polymer chain. According to the literature, anomeric hydrogens of saccharides present chemical shifts between δ 4.5 and δ 6.0 ppm (xylose - δ 4.4 ppm; glucose - δ 4.8 ppm (β) - δ 5.23 ppm (α) and mannose - δ 4.89 ppm (β)). In the positions between δ 3.0 and δ 4.5 ppm, overlapping signals from the other hydrogens of the sugars present in the polymer chain are observed; a low resolution spectrum is not observed at the coupling constants between the hydrogens of this part of the molecule. The singlet at δ 2.2 corresponds to the resonance signal of long-chain CH_2_ groups. The integration of these signals indicates that equivalent hydrogens are present in a relative proportion of 4:1, with respect to the anomeric hydrogen located at δ 5.23, assigned to the glucose residue. Signals are also observed due to the resonances of protons belonging to methyl groups of the polymer chain, confirming the results of chromatography. The presence of a spot indicative of correlation of scalar coupling in the 2D-COSY spectrum confirms the link from the polymer sample with its glycosidic portion (Figure 5). This result is consistent with the literature data that suggests that glycolipids or hetero-polysaccharide bioemulsifiers contain covalently linked hydrophobic side chains [33]. The low resolution and the great superposition of the signals observed in ^1^ H NMR spectra and confirmed by the COSY spectra suggest a high-molecular-weight bioemulsifier molecule with a polymeric pattern containing sugars and long chain aliphatic groups

## Conclusion

*T. mycotoxinivorans* CLA2 was able to produce a bioemulsifier from MM medium containing biodiesel residue adsorbed on diatomaceous earth particles. The optimization of the growth medium enables determination of the optimum range of bioemulsifier production and emulsifying activity. The microbial bioemulsifier appeared to be a lipid-polysaccharide complex composed of monosaccharides and long chain aliphatic groups with the ability to emulsify hydrocarbons and kerosene. Thus, the bioemulsifier is promising for the treatment of oily wastewater by accelerating biodegradation through facilitating emulsion formation. The biodiesel residue is a cheap substrate and is a very promising alternative for the low-cost production of bioemulsifiers by yeasts.

## Methods

### Bioemulsifier production and biodiesel refinery residue characteristics

A total of 30 yeast strains were screened for their ability to produce bioemulsifiers from biodiesel refinery wastes that consisted of diatomaceous earth impregnated with organic materials. The identities of the compounds adsorbed to the diatomaceous earth filter material were determined by energy-dispersive X-ray analysis (EDX), and they are reported in Table 3. The composition of the fatty acid methyl esters of the residues was determined by gas chromatography analysis, which indicated the presence of hexadecanoic acid (22. 92%), 9,12–octadecadienoic acid (36.74%), 9-octadecenoic acid (34.74%), and octadecanoic acid (5.60%). The yeast strains used in this study were previously isolated from effluents of the dairy industry [9]. The yeast strains were maintained in GYMP broth (2% (w/v) glucose, 0.5% yeast extract, 1% malt extract, and 0.2% NaH_2_PO_4,_ with 20% (v/v) glycerol added at 80°C.

**Table 3:** EDX analysis of the diatomaceous earth particles

**analyte**	**diatomaceous earth control**	**diatomaceous earth adsorbed**
SiO_2_	77.227%	57.936%
Al_2_SO_3_	18.505%	14.522%
K_2_O	1.877%	1.222%
Fe_2_SO_3_	1.371%	0.856%
P_2_O_5_	0.396%	0.302%
TiO_2_	0.376%	0.232%
SO_3_	0.107%	0.081%
BaO	0.116%	0.060%
ZrO_2_	0.016%	0.016%
CuO	0.005%	0.011%
Rb_2_O	0.005%	0.003%
Organic material	n.d	24.782%

n.d: not detected.

### Assay

The yeast strains were grown on Sabouraud dextrose agar medium (Difco, Detroit MI) for 24 h at 28°C. Subsequently, individual colonies were resuspended in 50-mL plastic tubes containing 20 mL of mineral minimal (MM) medium. The samples were homogenized in a vortex tube agitator for 5 minutes at maximum speed. After homogenization, samples were inoculated in Erlenmeyer flasks containing liquid MM medium. The optical densities (ODs) of the samples at 600 nm were measured, and these values were used to determine the volume to be transferred to test flasks to obtain an (OD) of 0.1. The bioemulsifier production assays were carried out in Erlenmeyer flasks containing 50 mL of (MM) medium with K_2_HPO_4_ (3.4 g), KH_2_PO_4_ (4.3 g), MgCl_2_.2H_2_O (0.3 g), (NH_4_)_2_SO_4_ (1 g), yeast extract (0.5 g), and 20 g/L biodiesel residue, which were incubated in an orbital shaker (200 rpm) at 28°C for 144 hours. After incubation, the yeast cells were separated from the growth medium by centrifugation at 5,000 rpm for 20 minutes at 4°C. Samples of cell-free medium were evaluated for emulsifying activity by the emulsification index (E_24_) as described by Cameron et al. [34]. In these assays, 4-mL aliquots of the cell-free filtrate were mixed with 6 mL of kerosene in a test tube and vortexed vigorously for 2 minutes. After 24 hours, the proportion of kerosene emulsified was compared with the total volume of kerosene added. The emulsification index (E_24_) was calculated using the following formula: height of the emulsion layer/total height X 100. To verify the stability of the emulsions, the emulsification index was also evaluated 4 weeks after the emulsion preparation, as described above. Tukey test with a significance level of 0.05 was applied for means comparison.

### Optimization of the bioemulsifier production and emulsifying activity

RSM was used to evaluate the best conditions for bioemulsifier production and emulsifying activity. This study was conducted by employing the statistical CCRD methodology. The central composite design with three factors and five levels, including six replicates at the center point, totaling 20 assays, was used for fitting a second order response surface. Equation 3 describes the regression model used for the factorial planning, including the interaction terms:

(3) $$Y=b_{0}+b_{1}X_{1}+b_{2}X_{2}+b_{3}X_{3}+b_{11}X_{1}^{2}+b_{22}X_{2}^{2}+b_{33}X_{3}^{2}+b_{12}X_{1}X_{2}+b_{23}X_{2}X_{3}+b_{13}X_{1}X_{3}$$

in which *b*o is the intercept term; *b1*, *b*2, and *b*3 are linear coefficients; *b*11, *b*22, and *b*33 are squared coefficients; and *b*12, *b*23, and *b*13 are interaction coefficients. Combinations of factors (such as *X*_1_*X*_2_) represent an interaction between the individual factors in that term. The response represents a function of the factor levels. *Y* is the predicted response variable, with *Y*_1_ referring to bioemulsifier production and *Y*_2_ referring to emulsifying activity (E_24_), and *X*_1_, *X*_2_ and *X*_3_ are the independent variables corresponding to the yeast extract, biodiesel residue and (NH_4_)_2_SO_4_ concentration, respectively.

RSM was applied to build a statistical model adjusted to the experimental conditions described and to optimize the conditions for the production of bioemulsifiers using the R statistical software (Version 2.8.1, 2008; Lucent Technologies).

The yeast strains were grown on Sabouraud dextrose agar medium (Difco, Detroit MI) for 24 hours at 28°C. Colonies were then resuspended in 50-mL plastic tubes containing 20 mL of MM medium. The samples were homogenized in a vortex tube agitator for 5 minutes at maximum speed. After homogenization, samples were inoculated in Erlenmeyer flasks containing liquid MM medium. The ODs of the samples at 600 nm were measured, and these values were used to determine the volume to be transferred to test flasks to obtain a final OD of 0.1.

The experiments were conducted in 250-mL Erlenmeyer flasks containing 100 mL of MM medium supplemented with biodiesel residue (carbon source), yeast extract (growth factors) and (NH_4_)_2_SO_4_ (nitrogen source), following the specifications in Table 4. The flasks were incubated at 28°C under continuous agitation at 200 rpm for up to 144 hours. For the recovery of the bioemulsifier, cells were removed from the culture broth by centrifugation at 5,000 rpm for 20 minutes at 4°C. The supernatant was filtered through a 0.45-μm Millipore membrane, and four volumes of cold ethanol were added. The white precipitate that formed was collected by centrifugation at 5,000 rpm for 20 minutes and treated with chloroform-methanol (2:1, v/v) to remove residual oils and was dried at 60°C to a constant mass. Samples of cell-free medium were also evaluated for emulsifying activity by the emulsification index (E_24_).

**Table 4:** Experimental range of the three variables studied using CCRD in terms of actual and coded factors

Variables	-1.68	-1	0	1	1.68
Yeast extract (g/L)	0.32	1	2	3	3.68
Biodiesel reidue (g/L)	6.4	20	40	60	73.6
(NH4)_2_SO_4_ (g/L)	0.31	1.26	2.66	4	5.02

### Emulsifying activity using different hydrophobic substrates

The ability of the bioemulsifier to emulsify some hydrophobic substrates was studied by replacing kerosene in the emulsification assay. The assays were conducted using the crude bioemulsifier at a concentration of 10 mg/mL in deionized water. Samples of 4 mL (emulsifier solution) were transferred to screw-cap tubes, and 6 mL of the each following hydrophobic substrates was added: hexane, octane cyclohexane hexadecane, toluene, xylene, gasoline or kerosene. The emulsifying activity was assessed by the emulsification index (E_24_). All assays were performed in triplicate. Tukey test with a significance level of 0.05 was applied for means comparison.

### Purification and partial characterization of the biochemical composition of the bioemulsifier

For purification, the crude water-soluble bioemulsifier was then applied to a Sephacryl S-200 column (1.6 x 57 cm, Pharmacia K 16/70 column) coupled to an FPLC system (Pharmacia). The column was pre-equilibrated with deionized water, and the products were eluted with 0.1 M degassed PBS buffer. Fractions (2 mL each) were collected, maintaining a flow rate of 1.0 min/mL and monitoring absorbance at 280 nm. Total sugars (by the phenol–sulfuric acid method), total protein (by the Bradford method) and emulsification activity (E_24_) were determined. The fractions with the highest emulsifying activities and carbohydrate concentration were pooled and lyophilized (Labconco Corporation, Kansas City, MO), and their lipid and carbohydrate composition was analyzed.

To determine the fatty acid composition of the lipid moiety of the bioemulsifier, approximately 0.5 mg of material (bioemulsifier) was placed in an airtight vial with a screw cap, dissolved in 1 mL of methanol P.A. (Merck), and stirred until homogeneity was reached. Approximately 50 μL of trifluoroacetic acid (Sigma) was added to this mixture. The resulting sample was placed in 2.5-mL microtubes and incubated at 120°C in a water bath for 18 hours. After the incubation, the solvent was completely evaporated using a nitrogen stream. Fatty acid methyl esters (FAMEs) were extracted three times with n-hexane and analyzed with a Varian model 3380 gas chromatograph using He as the carrier gas. A flame ionization detector (FID) and a CP-Sil 88 capillary column (50 m x 0.25 mm ID) were used. The column temperature was initially set at 170°C for 1 minutes and was programmed to rise linearly to 250°C at a rate of 4°C/min; the thermal program was halted isothermally at this temperature. The injector and detector block temperatures were 250°C. The fatty acid-methyl ester mixture (TM 37, FAME, Mix 47885; Supelco, USA) was used as the reference standard. To verify the presence of free fatty acids, these procedures were performed on samples of crude and pure bioemulsifiers that were not hydrolyzed with trifluoroacetic acid.

The carbohydrate composition of the bioemulsifier was determined by gas chromatography and mass spectrometry. A lyophilized sample of the bioemulsifier (1 mg) was hydrolyzed in a sealed tube with 150 μL of 2 M trifluoroacetic acid (CF_3_COOH) at 120°C for 4 hours. After evaporation, the residue was washed twice with methanol; the sample was then reduced with 1 M aqueous sodium borohydride (NaBH_4_, 100 μL) and acetylated with a mixture of potassium acetate (100 μg) and acetic anhydride (100 μL) at 100°C for 2 hours. The excess reagent was removed by evaporation, and the sample was washed several times with ethanol. The alditol acetates were extracted with ethyl acetate and water (1:1, v:v) and analyzed using a GC–MS (SHIMADZU, model QP 5050 A) equipped with a PTE-5-Supelco (30 m x 0.25 mm ID, 0.25 μm film) column using He as the carrier gas. The column temperature was programmed to increase from 100°C (1 minute) to 200°C at a rate of 4°C/min, followed by a 20°C/min increase to 300°C, and the column was maintained at this temperature for 5 minutes. These procedures were performed on crude and pure bioemulsifier samples that were not hydrolyzed to verify the presence of free carbohydrates in these samples. Sugars were identified by comparing the relative retention times of sample peaks with standards. The following sugar standards were purchased from Supelco, USA: glucose, mannose, galactose, rhamnose, fucose, ribose, arabinose, and xylose. The results were recorded and processed using the Class 3.02 software (Shimadzu) and expressed as relative peak areas for each sugar.

### Characterization of the bioemulsifier by Nuclear Magnetic Resonance (^1^ H NMR)

NMR spectra were recorded using a Bruker DRX400 *AVANCE* spectrometer operating at 400 MHz and equipped with an inverse detection (*ϕ=*5 mm) probe with a z-gradient coil, using deuterated water (D_2_O) as the solvent. The NMR analyses were carried out at 27°C (300 K). One-dimensional ^1^ H NMR spectra were obtained with water suppression. Bi-dimensional ^1^ H homonuclear correlation spectroscopy (COSY) was used to identify the pattern of hydrogen signal assignments [35]. The results obtained with 1D and 2D NMR spectral methods were utilized to characterize the bioemulsifier molecules.

## Abbreviations

RSM = Response surface methodology; 1H NMR = Proton nuclear magnetic resonance; SACs = Surface-active compounds; COSY = Correlated spectroscopy; E24) = Emulsifying activity in 24 h; CV = Coefficient of variation; CCRD = Central composite rotatable design; R2 = Determination coefficient; δ = Anomeric hydrogen; ppm = Parts per million; GC–MS = Gas chromatography–mass spectrometry; p = Probability; FAME = Fatty acid-methyl ester.

## Competing interests

The authors declare that they have no competing interests.

## Authors’ contributions

ASM conducted experiments using Response Surface Methodology and analyzed the results. VSD conducted assay growth microbial and purification of bioemulsifier. MVDS reviewed the manuscript. IL Conducted the characterization of the bioemulsifier by nuclear magnetic resonance (^1^H NMR). DBG conducted Response Surface Methodology (RSM), analyzed the results and reviewed the manuscript. EPS conducted partial characterization of the biochemical composition of the bioemulsifier by gas chromatograph. VLS analyzed the results and reviewed the manuscript; MM: minimum media; ODs: optical densities. All authors read and approved the final manuscript.
